# Skull fracture and epidural hematoma caused by use of a Mayfield skull clamp in an adult patient with chronic hemodialysis: a case report

**DOI:** 10.1186/s13256-021-02776-8

**Published:** 2021-04-09

**Authors:** Takeo Furuya, Masashi Yamazaki, Tetsuharu Nemoto, Akihiko Okawa, Seiji Ohtori

**Affiliations:** 1 Department of Orthopaedic Surgery, Graduate School of Medicine, Chiba University, 1-8-1 Inohana, Chuo-Ku, Chiba-shi, Chiba 260-8677 Japan; 2 Department of Orthopaedic Surgery, University of Tsukuba, 1-1-1 Tenodai, Tsukuba-shi, Ibaraki 305-8575 Japan; 3 Department of Orthopaedic Surgery, Seirei Sakura Citizen Hospital, 2-36-2 Ebaradai, Sakura-shi, Chiba 285-8765 Japan

**Keywords:** Mayfield skull clamp, Skull fracture, Epidural hematoma, Adult patient, Case report

## Abstract

**Background:**

Mayfield skull clamps are widely used and indispensable in current neurosurgery. Complications such as skull fractures or intracranial hematoma from using a Mayfield skull clamp have largely been reported in the pediatric population, are likely related to the relative thinness of the skull, such as in patients with hydrocephalus, and are extremely rare in adults. Here, we report a case of skull fracture and epidural hematoma caused by a Mayfield skull clamp used for posterior decompression surgery in an adult patient with chronic hemodialysis.

**Case presentation:**

A 67-year-old Asian male patient with a history of dialysis-dependent chronic renal failure over 36 years suffered from severe cervical myelopathy. Neurological examination and radiographic images revealed cervical spondylotic myelopathy due to dialysis-related spondyloarthropathy. Laminoplasty was planned on patient consent. A Mayfield skull clamp was applied with the patient supine. Torque was applied to the screws with gentle care, but there was no resistance and it was not easy to reach the standard 60 lb (267 N) to 80 lb (356 N). Because a skull fracture was suspected, we canceled the surgery. Emergency head computed tomography showed depressed skull fractures underlying the single-pin sites with an associated epidural hematoma. The fractures and epidural hematoma were treated conservatively, and spontaneous resolution of the hematoma was confirmed. Cervical laminoplasty was performed successfully using a mask-type head holder on the subsequent day.

**Conclusions:**

As a precaution for fractures and epidural hematoma in neurosurgical patients with bone fragility or a thin skull, use of a mask-type fixing device or halo ring is recommended.

## Background

Three-pronged head holders such as a Mayfield skull clamp (Integra LifeSciences, Cincinnati, Ohio, United States of America) provide rigid cranial fixation during intracranial and spinal surgeries. Surgeons can stabilize the surgical field effectively by using head holders. Three-pronged head holders also protect the face and the eyes from physical compression in the prone position. However, a head holder can sometimes, although rarely, cause serious complications. Here, we report skull fractures and an epidural hematoma caused by Mayfield skull clamp used for posterior cervical decompression in an adult patient with chronic hemodialysis. We also review previous reports of similar cases and alert surgeons to this complication.

## Case presentation

**Fig. 1:**
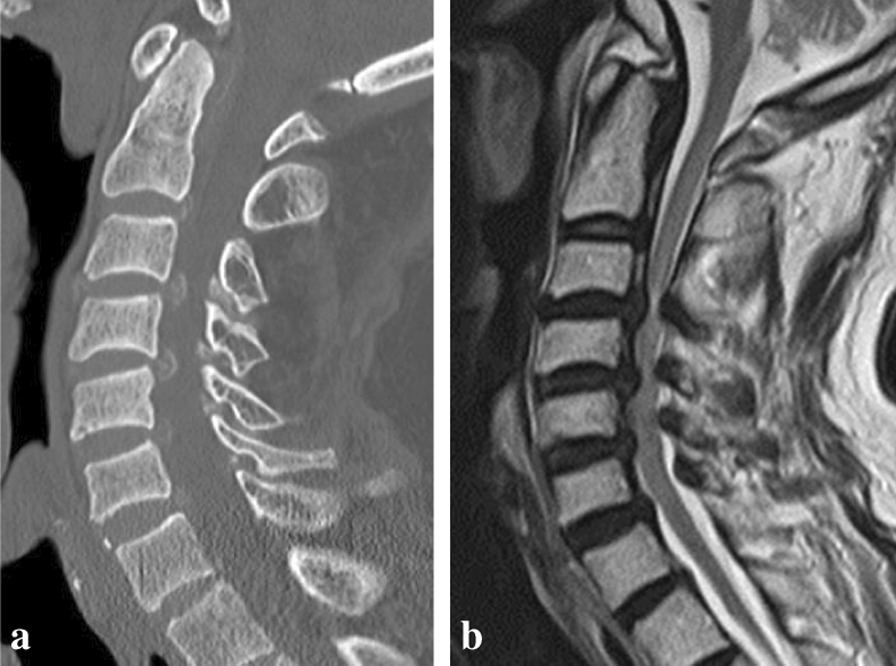
**a** Computed tomography (CT) showing an extradural amyloid deposit and hypertrophied ligament, which are characteristic findings for dialysis-related spondyloarthropathy. **b** Magnetic resonance imaging (MRI) showing multilevel spinal cord compression at the C3–C6 levels and T2-weighted hyperintensity of the spinal cord at the C3–C4 level

A 67-year-old Asian man was admitted to our hospital with a complaint of progressive myelopathy. Over the preceding several months, gait disturbance and numbness in his upper extremities had progressed rapidly. He had a medical history of dialysis-dependent chronic renal failure over 36 years. He also had a medical history of hypertension for 17 years and parathyroidectomy for parathyroiditis 6 years ago. He had no history of diabetes. He also had no tobacco smoking or alcohol history. He has no history of cancer or similar condition in his family. His family consisted of his wife and daughter. With regard to his social and professional history, he had originally worked at a desk but had already retired. He was taking a selective angiotensin 1 (AT1) receptor blocker as an antihypertensive drug, as well as a proton pump inhibitor and sodium alginate as gastrointestinal drugs. He was also taking zolpidem tartrate tablets as a sleep aid, sennoside as constipation medicine, and nonsteroidal antiinflammatory drugs (NSAIDs) as a painkiller. Vital signs on admission were as follows. His blood pressure was 145/70 mmHg. His pulse was 65 beats/minute. His body temperature was normal at 36.8°C. Oxygen saturation was 98% on room air. There were no abnormal findings in the head, and consciousness level on the Glasgow Coma Scale was E4V5M6. The heart was in regular rhythm with no murmurs. Lung auscultation was clear with no distress. The abdomen was soft with no tenderness. No abnormalities were found on the body surface of the extremities and trunk, except for a dialysis shunt placed in the right elbow. The patient had difficulty handling chopsticks and fastening shirt buttons. Ten-second grip and release test showed a decrease in hand dexterity, with 15 and 16 repetitions on the right and left, respectively (normal values are more than 25 repetitions). Manual muscle testing showed grade 3/5 muscle weakness in finger extension, finger flexion, and finger abduction on the right. Other muscle strength was normal at grade 5/5. The patient had a dialysis shunt in the right upper extremity. He had numbness and sensory loss in the C6 area of his right upper limbs. Deep tendon reflexes were normal in the upper extremities, and the patella tendon reflex showed hyperactivity. He could not walk without a cane and needed a handrail to go up and down stairs. Neurological testing demonstrated motor weakness of right upper limbs, showing grade 3/5 muscle strength. He had numbness and sensory loss in his right upper limbs. Deep tendon reflexes were normal in the upper extremities and showed hyperactivity in the lower extremities. The lower extremities showed spasticity. The Japanese Orthopaedic Association (JOA) score excluding bladder function was 9.5 points (full score is 14.0 points). Blood tests (Table 1) showed normal values of Ca 8.8 mg/dL and P 3.9 mg/dL. Level of β2-microglobulin was slightly higher at 25.6 mg/L. Intact parathyroid hormone (intact-PTH) was 62 pg/mL, which is normal. Serology and microbiology tests were normal. Though radiological examination did not show severe destructive changes in the cervical spine, computed tomography (CT) detected extradural amyloid deposits and a hypertrophied ligament, which are characteristic findings for dialysis-related spondyloarthropathy (Fig. 1a). Magnetic resonance imaging (MRI) showed multilevel spinal cord compression at the C3–C6 and T2-weighted hyperintensity of the spinal cord at the C3–C4 level (Fig. 1b). Bone mineral density by dual X-ray absorptiometry (DXA) was 0.799 g/cm^2^ for the femoral neck and 1.213 g/cm^2^ for the lumbar spine. These were 84% of the young adult mean (YAM) for the femoral neck and 102% of the YAM for the lumbar spine. Based on the history and clinical findings, we diagnosed cervical spondylotic myelopathy due to dialysis-related spondyloarthropathy. The patient consented to undergo cervical laminoplasty.

**Table 1:** Laboratory data on admission

Parameter	Value	Parameter	Value
Na	132 mEq/L	WBC	9.1 × 10^3^/μL
K	3.3 mEq/L	RBC	3.32 × 10^6^/μL
Cl	95 mEq/L	Hb	10.3 g/dL
Ca	8.8 mg/dL	Hct	30.80%
P	3.9 mg/dL	Plt	159 × 10^3^/μL
BUN	28 mg/dL		
Cre	7.48 mg/dL	β2-microglobulin	25.6 mg/L
T. pro	5.8 g/dL	Intact-PTH	62 pg/mL
Alb	3.4 g/dL		
T. bil	0.4 mg/dL		
D. bil	0.1 mg/dL		
AST	24 U/L		
ALT	26 U/L		
ALP	125 U/L		
γ-GTP	24 U/L		
LDH	258 U/L		
CPK	455 U/L		
CRP	0.1 mg/dL		
Glu	114 mg/dL		

*BUN* blood urea nitrogen, *Cre* creatinine, *T.pro* total protein, *Alb* albumin, *T. bil* total bilirubin, *D. bil* direct bilirubin, *AST* aspartate aminotransferase, *ALT* alanine aminotransferase, *ALP* alkaline phosphatase, *γ-GTP* γ-glutamyl transpeptidase, *LDH* lactate dehydrogenase, *CPK* creatine phosphokinase, *CRP* C-reactive protein, *GLU* glucose, *WBC* white blood cell count, *RBC* red blood cell count, *Hb* hemoglobin, *Hct* hematocrit, *Plt* platelet count, *Intact-PTH* intact parathyroid hormone

**Fig. 2:**
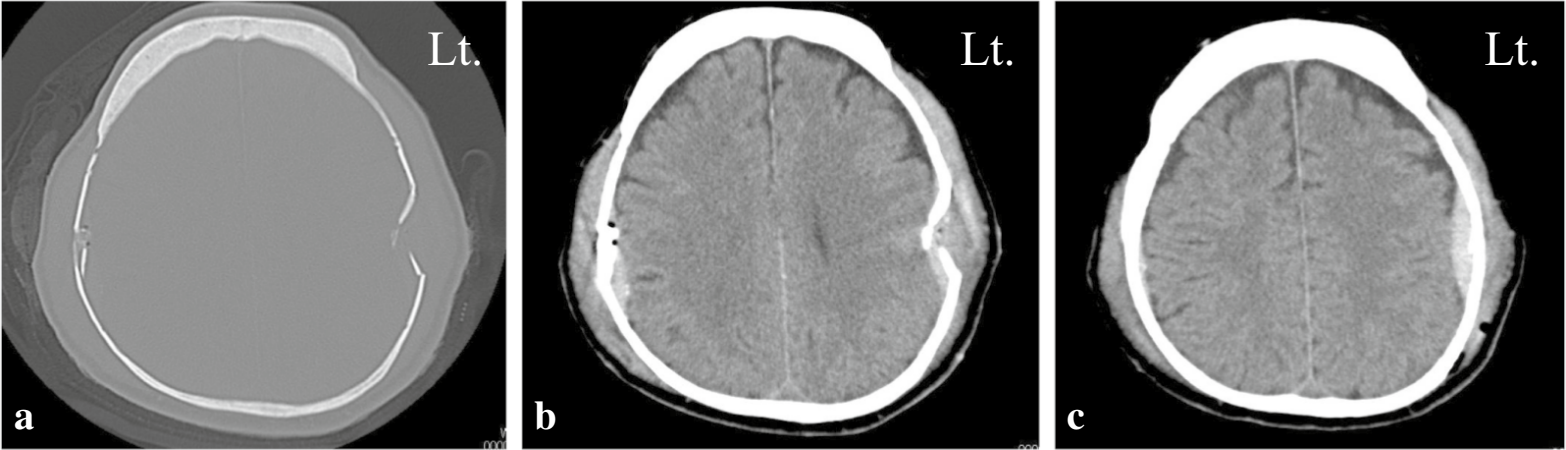
Emergency head computed tomography (CT) showing depressed skull fractures underlying the single-pin sites (**a**, **b**) with an associated epidural hematoma on the left (Lt.) side (**c**)

**Fig. 3:**
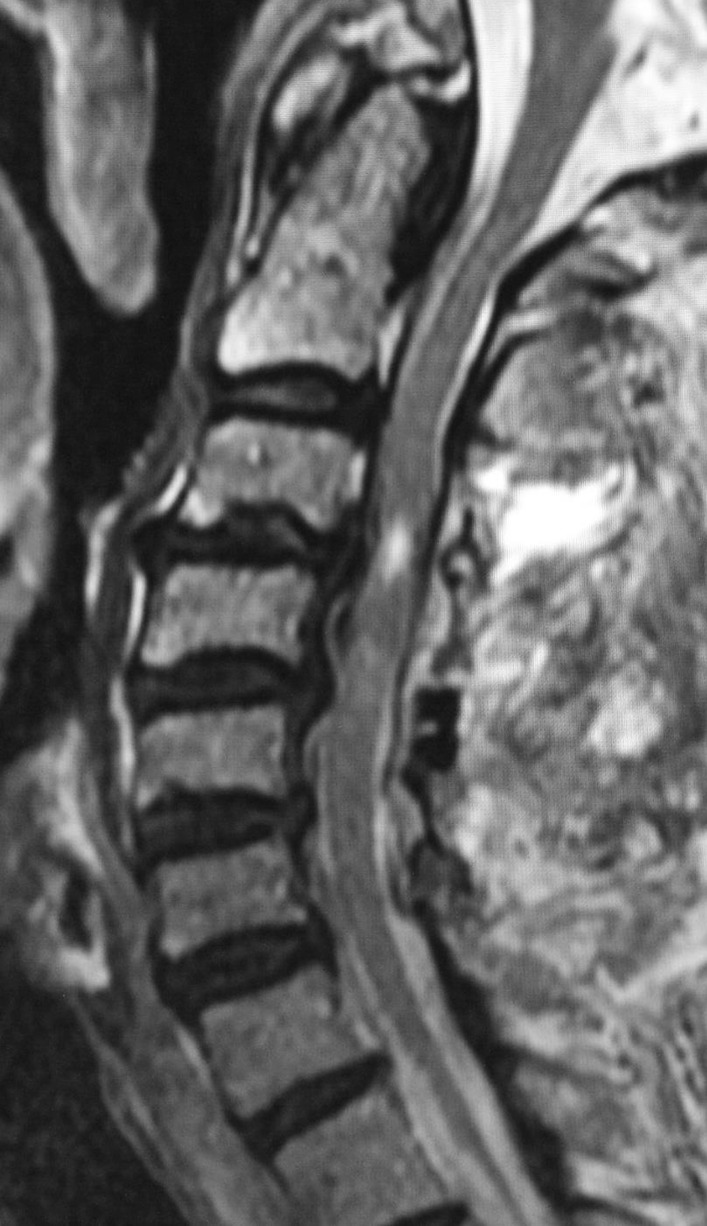
Postoperative magnetic resonance imaging (MRI) confirming successful decompression of the spinal cord

After inducing general anesthesia, a Mayfield skull clamp was applied with the patient supine. Torque was applied to the screws with gentle care, but there was no resistance, and it was not easy to reach the standard 60 lb (267 N) to 80 lb (356 N) torque. Initially, we attributed the lack of torque to the failure of the Mayfield skull clamp itself. Another attempt was made with an alternative fixator. To change the insertion point, the left and right sides of the instrument were reversed. Nevertheless, we could not stabilize the skull and obtain appropriate torque. A skull fracture was suspected, so we canceled the surgery. After the patient awakened from anesthesia, we confirmed that no cranial nerve symptoms appeared. An emergency postoperative head CT showed depressed skull fractures underlying the single-pin sites (Fig. 2a, b) with an associated epidural hematoma on the left side (Fig. 2c). We immediately consulted the neurosurgery department. Conservative treatment with rest, blood pressure control, and suspension of antiplatelet medication during dialysis was proposed. If neurological symptoms appeared, surgery was also suggested. We decided to treat the fractures and epidural hematoma conservatively. Spontaneous resolution of the hematoma was confirmed with no appearance of neurological symptoms. The patient was temporarily discharged 10 days after the initial surgery. Cervical laminoplasty was performed successfully using a mask-type head holder on the subsequent day (Fig. 3). After the decompression surgery of the cervical spine, the symptoms of cervical myelopathy showed a consistent improvement. He is now able to use chopsticks, which he could not do before the surgery, and his lower limb spasticity is reduced, making walking easier. His JOA score improved to 10.0 points for 1.5 years at the time of the last follow-up.

## Discussion and conclusions

We report a case of skull fracture and epidural hematoma caused by a Mayfield skull clamp used for spine surgery in an adult patient. Complications arising from the use of a Mayfield skull clamp have been largely reported in the pediatric population, but adult cases are rare. This case is characterized by a long history of hemodialysis and a thin skull.

Although three-pronged head holders such as a Mayfield skull clamp are safe and useful surgical instruments for intracranial and cervical spine surgeries, care should be taken because they can sometimes cause serious complications. The recommended screw force for a Mayfield skull clamp lies between 60 and 80 lb for adult patients, and between 40 and 60 lb for children younger than 15 years [1]. The conical shape of the pins and the pressure-adjusting mechanism in a Mayfield skull clamp make it highly unlikely to penetrate the bone [2]. Chavasiri *et al.* examined skull thickness at the area of halo pin insertion [3]. They noted the thickness of the skull at the halo pin insertion site increases gradually with age. The average thickness of the skull at the anterolateral aspect was 5.53 mm (0–9 years) to 8.79 mm (80–89 years), and the average thickness of the skull at the posterolateral aspect was 5.45 mm (0–9 years) to 8.95 mm (80–89 years). An inner table of a 2 mm thick skull bone was completely penetrated at a screw torque of 160 lb [4]. From those studies, we conclude that a normal skull is unlikely to fracture at a screw torque of 80 lb. In this case, the thickness of the skull measured by postoperative CT was 1.7 mm around the pin insertion site, which was very thin.

To date, complications arising from the use of a Mayfield skull clamp have been largely reported in the pediatric population. Complications include depressed skull fracture, epidural hematoma, cerebrospinal fluid (CSF) leak, subdural hematoma, venous air embolism, pin site infections, head slippage, and scalp lacerations [5–7]. Skull fractures and intracranial hematoma in the pediatric population are likely related to the relative thinness of the skull, such as in patients with hydrocephalus. By contrast, their occurrence in adults is extremely rare. Complications of skull fracture and epidural hematoma as a consequence of using a pin-type head holder skull clamp in an adult were first reported by Erbayraktar in 2001 [8]. Sade *et al.* reported the first case in an adult as a consequence of using a Mayfield skull clamp [9]. To our knowledge, only nine cases have been reported thus far, including our case (Table 2) [8–16].

**Table 2:** Skull fractures and intracranial hemorrhages by using pin-type head holder in adult patients

Case	Authors	Reported year	Age (years)/gender	Diagnosis	Surgical plan	Name of head holder	Application pressure	Complications	Management of complications	Risk factors
1	Erbayraktar *et al.* [8]	2001	23/M	Pituitary adenoma	Tumor excision	Multipoise	N/A	Skull fracture and EDH	Evacuation of EDH	Instrument failure (misplaced spring)
2	Sade and Mohr [9]	2005	24/M	Meningioma (brain)	Tumor excision	Mayfield	N/A	Skull fracture and EDH	Evacuation of EDH	Thin calvarium (chronic compression by SOL)
3	Jha *et al.* [10]	2009	22/M	Fourth ventricular tumor	Tumor excision	Mayfield	60	Skull fracture and EDH	Evacuation of EDH	Thin calvarium (chronic hydrocephalus)
4	Lee* et al.* [11]	2010	38/F	Postoperative pseudoarthroses	Cervical foraminotomies and instrumented fusion	Mayfield	60	Skull fracture and EDH	Evacuation of EDH	Thin calvarium (unknown reason)
5	Naik V *et al.* [12], Bindra *et al.* [13]	2012	40/F	Schwannoma (brain)	Tumor excision	Mayfield	60	EDH (bilateral)	Evacuation of EDH	N/A
6	Matouk *et al.* [14]	2012	79/M	CSM	Posterior cervical decompression	Mayfield	#	Skull fracture and EDH	Conservative	Thin calvarium (dialysis-dependent chronic renal failure), antiepileptics
7	Won *et al.* [15]	2012	61/F	Intracerebral hematoma	Removal of hematoma	Fischer stereotactic head frame	N/A	Skull fracture, EDH, and increased volume of intracerebral hematoma	Evacuation of EDH	Inadvertent application
8	Haldar *et al.* [16]	2017	25/M	Glioma (brain)	Tumor excision	Sugita frame (four-pin)	N/A	EDH	Evacuation of EDH	N/A
9	Present case	2020	68/M	CSM	Laminoplasty	Mayfield	#	Skull fracture and EDH	Conservative	Thin calvarium (dialysis-dependent chronic renal failure)

*CSM* cervical spondylotic myelopathy, *EDH* epidural hematoma, *SOL* space-occupying lesion, *M* male, *F* female, *N/A* not applicable

^#^appropriate pressure could not be maintained

The risk factor in the present case is considered to be long-term dialysis. Matouk *et al.* reported a case similar to ours [10]. They reported a case of a patient with a combination of dialysis-dependent chronic renal failure and long-standing use of antiepileptic drugs that contributed to pathological fracture of the skull vault during routine use of a Mayfield skull clamp. A long history of dialysis has been a factor strongly associated with femoral neck fractures [17]. The incidence of hip fractures in a population of patients who undergo hemodialysis was 17.4 times greater than in the general population [18]. In our patient, bone mineral density assessed by DXA was normal. However, fracture risk is considered to depend on bone quality rather than bone mineral density, and DXA measurement of bone mineral density is not very useful to predict fracture.

To prevent this complication, we suggest some strategies. In cervical spine surgery, preoperative evaluation by plain CT or CT myelogram is often performed. In cases where there is a possible risk of skull fracture, extending the scope of the CT scan to the head and preoperative evaluation and recognition of the thickness of the skull may be an option. Some modifications of head fixation have been reported. When using a Mayfield skull clamp in cases where bone cortex thinning and bone fragility are suspected, careful placement and gentle tightening is proposed. It is recommended to apply the torque force slowly and manually without applying axial pressure. Aoki and Sakai describe using a rubber cup from a medical bottle over the skull pins as a barrier to the pins penetrating too deeply [19]. Another option is to decrease the torque on the screws to under 60 lb even in adults. However, there is a possibility of decreased stability. By using Sugita frame, the pin insertion position can be set in the frontal region of the head, which is relatively thick. Another option considered as a useful alternative is to use a halo ring, increase the number of inserted pins, and reduce the torque on individual pins. Use of a mask-type or horseshoe-type head holder to stabilize the head and provide a rigid immobilization has also been recommended. We performed the reoperation using a mask-type head holder according to this recommendation and were able to complete the operation safely.

Skull fracture and epidural hematoma from use of a Mayfield skull clamp are extremely rare complications in adults. However, surgeons must be aware of the risk of these complications in patients with bone fragility or a thin skull. As a precaution for these complications, use of a mask-type or halo-ring fixing device is recommended.

## Data Availability

Not applicable.

## Abbreviations

CSF: Cerebrospinal fluid
CT: Computed tomography
DXA: Dual X-ray absorptiometry
Intact-PTH: Intact parathyroid hormone
MRI: Magnetic resonance imaging
NSAIDs: Nonsteroidal antiinflammatory drugs
Selective AT1 receptor blocker: Selective angiotensin 1 receptor blocker
JOA: Japanese Orthopaedic Association
YAM: Young adult mean

## References

[1] Yan HJ. Epidural hematoma following use of a three-point skull clamp. J Clin Neurosci. 2007;14:691–3. 10.1016/j.jocn.2006.02.012 17462901

[2] Medina M, Melcarne A, Musso C, *et al.* Acute brain swelling during removal of supratentorial cystic lesion caused by contralateral extradural hematoma: case report. Surg Neurol. 1997;47:428–31. 10.1016/s0090-3019(96)00243-1 9131023

[3] Chavasiri C, Chavasiri S. The thickness of skull at the halo pin insertion site. Spine. 2011;36:1819–23. 10.1097/BRS.0b013e3181d3cfa3 21099739

[4] Letts M, Kaylor D, Gouw G. A biomechanical analysis of halo fixation in children. J Bone Jt Surg 1988;70-B:277–9. 10.1302/0301-620X.70B2.3346303 3346303

[5] Baerts WD, de Lange JJ, Booij LH, *et al.* Complications of the Mayfield skull clamp. Anesthesiology. 1984;61:460–1. 10.1097/00000542-198410000-00017 6486508

[6] Vitali AM, Steinbok P. Depressed skull fracture and epidural hematoma from head fixation with pins for craniotomy in children. Childs Nerv Syst. 2008;24:917–23. 10.1007/s00381-008-0621-9 18389258

[7] Berry C, Sandberg DI, Hoh DJ, *et al.* Use of cranial fixation pins in pediatric neurosurgery. Neurosurgery. 2008;62:913–8. 10.1227/01.neu.0000318177.95288.cb 18496197

[8] Erbayraktar S, Gokmen N, Acar U. Intracranial penetrating injury associated with an intraoperative epidural haematoma caused by a spring-laden pin of a multipoise headrest. Br J Neurosurg. 2001;15:425–8. 10.1080/02688690120000000 11708547

[9] Sade B, Mohr G. Depressed skull fracture and epidural haematoma: an unusual post-operative complication of pin headrest in an adult. Acta Neurochir (Wien). 2005;147:101–3. 10.1007/s00701-004-0348-7 15455215

[10] Jha NK, Ebrahim S, Fallah A, *et al.* Pin-site epidural hematoma in an adult case of chronic hydrocephalus with associated thinning of the cranium. Br J Neurosurg. 2009;23:211–2. 10.1080/02688690802434830 19306183

[11] Lee MJ, Lin EL. The use of the three-pronged Mayfield head clamp resulting in an intracranial epidural hematoma in an adult patient. Eur Spine J. 2010;19(Suppl. 2):S187–9. 10.1007/s00586-010-1323-z PMC2899644 20155477

[12] Naik V, Goyal N, Agrawal D. Pin site bilateral epidural hematoma—a rare complication of using Mayfield clamp in neurosurgery. Neurol India. 2011;59:649–51. 10.4103/0028-3886.84368 21891964

[13] Bindra A, Rath GP, Chowdhury T, *et al.* Epidural hematoma at skull pin fixation sites may cause refractory intraoperative brain bulge. J Clin Anesth. 2012;24:509–10. 10.1016/j.jclinane.2011.09.005 22986325

[14] Matouk CC, Ellis MJ, Kalia SK, *et al.* Skull fracture secondary to application of a Mayfield skull clamp in an adult patient: case report and review of the literature. Clin Neurol Neurosurg. 2012;114:776–8. 10.1016/j.clineuro.2011.12.036 22261664

[15] Won YD, Kim CH, Cheong JH, *et al.* Skull perforation and depressed fracture following skull fixation for stereotactic surgery. Korean J Neurotrauma. 2012;8:48–50.

[16] Haldar R, Kumar T, Misra G. Extradural hemorrhage secondary to skull pin fixation manifesting as intractable intraoperative brain swelling. J Neurosurg Anesthesiol. 2017;29:375–6. 10.1097/ANA.0000000000000330 27341671

[17] Alem AM, Sherrard DJ, Gillen DL. Increased risk of hip fracture among patients with end-stage renal disease. Kidney Int. 2000;58:396–9. 10.1046/j.1523-1755.2000.00178.x 10886587

[18] Coco M, Rush H. Increased incidence of hip fractures in dialysis patients with low serum parathyroid hormone. Am J Kidney Dis. 2000;36:1115–21. 10.1053/ajkd.2000.19812 11096034

[19] Aoki N, Sakai T. Modified application of three-point skull clamp for infants. Neurosurgery. 1989;25:660–2. 10.1097/00006123-198910000-00028 2677825

